# Parents’ experiences of an antenatal visit being part of a home visiting program in deprived areas

**DOI:** 10.1093/eurpub/ckac131.469

**Published:** 2022-10-25

**Authors:** G Lönnberg, K Edvardsson, J Leissner, R Salari, G Warner, A Sarkadi

**Affiliations:** Department of Public Health and Caring Sciences, Uppsala Universitet, Uppsala, Sweden

## Abstract

**Background:**

There are considerable health divides between residential areas in many Swedish cities. In more disadvantaged areas children grow up with poorer health outcomes than the country average. To meet the greater needs of children growing up in these areas through proportionate universalism, an extended home visiting program has been delivered. A novel part of this program has been the social worker and nurse later conducting home visits meeting the parents at the maternity care clinic before childbirth. The aim of this study was to explore parents’ experiences of that antenatal visit.

**Methods:**

Semi-structured interviews were carried out with nine mothers and three fathers around 3 months postpartum. Nine of the participants were foreign-born and a translator was used for four of the interviews. The interviews were recorded and transcribed verbatim and the data was analyzed with thematic analysis with an inductive approach.

**Results:**

The parents’ overall experiences are comprised in the main theme: ‘A feeling of security and care for the whole family'. This main theme is derived from the three following themes: 1) ‘Staff - a trustworthy source of information'. Parents perceived the staff as experienced and knowledgeable and appreciated obtaining information about practical things and about the Swedish system; 2) ‘Access to emotional support'. Several parents expressed the need for emotional support and valued that by meeting the staff they knew they had someone to turn to; 3) ‘Becoming familiar with the staff'. It was appreciated to know who will come to your home as this gave parents an increased sense of security.

**Conclusions:**

Initiating the program through introducing home visiting staff at a scheduled antenatal visit benefited parents, by giving them useful information and social support. The visit also seems to be indirectly beneficial as it contributes to building trust for the staff and the rest of the program.

**Key messages:**

• Initiating an extended home visiting program at a scheduled antenatal visit benefited parents, by giving them useful information and social support.

• Initiating an extended home visiting program at a scheduled antenatal visit contributed to building parents’ sense of trust for the staff and the rest of the program.

